# Development and Validation of an IL6/JAK/STAT3-Related Gene Signature to Predict Overall Survival in Clear Cell Renal Cell Carcinoma

**DOI:** 10.3389/fcell.2021.686907

**Published:** 2021-09-29

**Authors:** Chuanchuan Zhan, Chao Xu, Jiajun Chen, Chong Shen, Jinkun Li, Zichu Wang, Xiangrong Ying, Zhengang Luo, Yu Ren, Gangfeng Wu, Haojie Zhang, Manfei Qian

**Affiliations:** ^1^Shaoxing People’s Hospital, Shaoxing, China; ^2^Zhongshan Hospital, Xiamen University, Xiamen, China

**Keywords:** prognosis, IL6-JAK-STAT3, nomogram, WGCNA, renal cell carcinoma, immune

## Abstract

**Background:** Traditional clinicopathological features (TNM, pathology grade) are often insufficient in predictive prognosis accuracy of clear cell renal cell carcinoma (ccRCC). The IL6-JAK-STAT3 pathway is aberrantly hyperactivated in many cancer types, and such hyperactivation is generally associated with a poor clinical prognosis implying that it can be used as a promising prognosis indicator. The relation between the IL6-JAK-STAT3 pathway and ccRCC remains unknown.

**Methods:** We evaluated the levels of various cancer hallmarks and filtered out the promising risk hallmarks in ccRCC. Subsequently, a prognosis model based on these hallmark-related genes was established *via* weighted correlation network analysis and Cox regression analysis. Besides, we constructed a nomogram based on the previous model with traditional clinicopathological features to improve the predictive power and accuracy.

**Results:** The IL6-JAK-STAT3 pathway was identified as the promising risk hallmarks in ccRCC, and the pathway-related prognosis model based on five genes was built. Also, the nomogram we developed demonstrated the strongest and most stable survival predictive ability.

**Conclusion:** Our study would provide new insights for guiding individualized treatment of ccRCC patients.

## Introduction

Renal cell carcinoma (RCC), 1 of the 10 most common cancers in men and women with an increasing incidence since the 1990s, was expected to cause approximately 76,080 new cases and 13,780 deaths in 2021^1^. Among RCC, clear cell renal cell carcinoma (ccRCC) accounts for almost 75% of detected cases (Hsieh et al., 2017), imposing a significant burden on patients, families, and society. Primary tumor resection is the standard treatment (Subramanian and Haas, 2018) for clinically localized ccRCC. For advanced ccRCC, molecularly targeted drugs and mammalian rapamycin inhibition are the first-line treatments (Powles et al., 2018), but the therapeutic impact is not promising due to the tumor microenvironment being highly dynamic, adaptive, and heterogeneous (Heidegger et al., 2019). Patients greatly suffered as a result of this embarrassing situation. Therefore, in addition to traditional indicators (TNM, pathology grade, etc.), a more stable and robust improved prognosis method is desperately required.

The IL6-JAK-STAT3 pathway is always aberrantly hyperactivated in many cancer types (Wang and Sun, 2014; Zhang et al., 2016; Chiang et al., 2020; Pan et al., 2020), and this phenomenon is often associated with poor outcomes (Kusaba et al., 2005; Macha et al., 2011; Chen et al., 2013). This may be linked to a pathway that promotes tumor growth, affects tumor microenvironment, and induces antitumor immunity (Johnson et al., 2018). Given that immune checkpoint inhibition has significantly enhanced the treatment of patients with advanced cancer, numerous studies (Austin et al., 2014; Atsaves et al., 2017; Bu et al., 2017) have explored the relationship between them. The results demonstrated that signaling *via* the IL6-JAK-STAT3 pathway induces PD-1 and/or PD-L1 expression. However, no IL6-JAK-STAT3 pathway-related method has been published to date to predict ccRCC prognosis.

**FIGURE 1:**
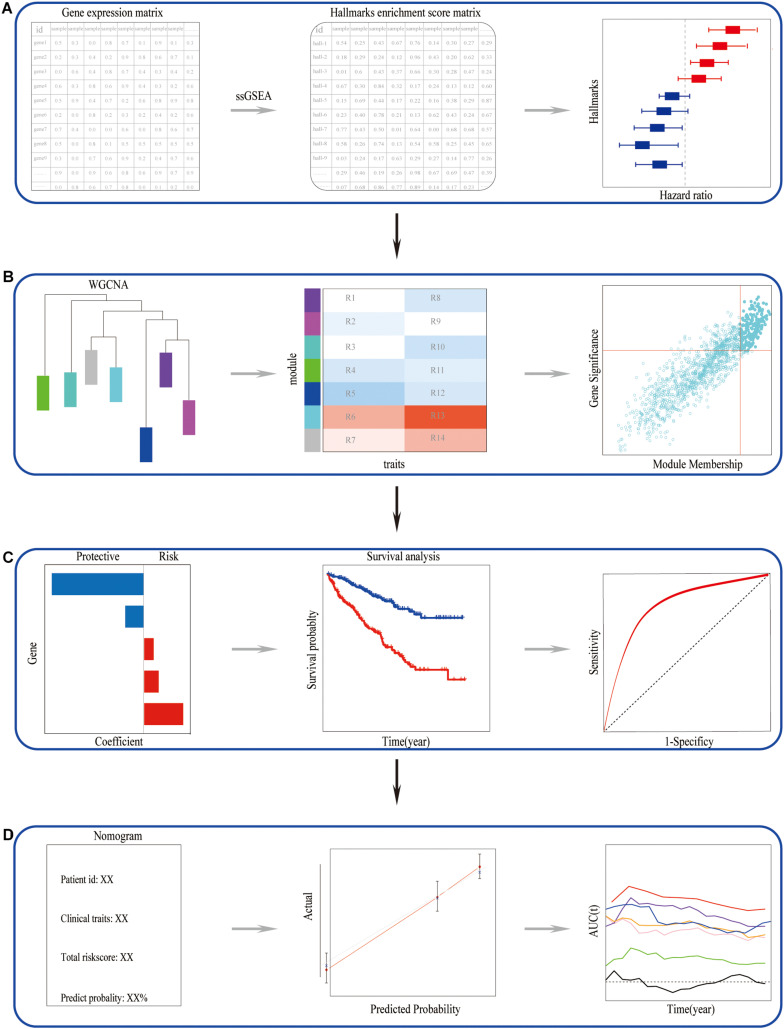
The main flowchart of the study. **(A)** Identified the candidate risk hallmarks in ccRCC. **(B)** Picked out the genes closely related to the candidate risk hallmarks. **(C)** Constructed and validated a candidate risk hallmarks-related signature. **(D)** A nomogram can perform well in predictive prognosis in ccRCC.

This study first identified the IL6-JAK-STAT3 pathway among various cancer hallmarks as a promising risk hallmark in ccRCC. Secondly, a scare-free network was constructed, and the genes significantly correlated with the IL6-JAK-STAT3 pathway in the network were picked out. Subsequently, IL6-JAK-STAT3 pathway-related gene signature was established. Besides, we explored the correlation of alternative polyadenylation of these genes and ccRCC prognosis as well as the IL6-JAK-STAT3 pathway and tumor microenvironment. Finally, a nomogram based on risk gene signature with traditional indicators was developed to improve the predictive power and accuracy. The main flowchart of the study was shown in Figure 1.

## Materials and Methods

### Dataset Preparation and Data Processing

This research included 640 ccRCC tumors at various stages and 72 normal kidney tissues with associated clinical data. RNA-seq data of 539 ccRCC and 72 normal kidney tissue used as training and inside validation cohort were obtained from The Cancer Genome Atlas (TCGA). The microarray dataset GSE40435 was downloaded from NCBI GEO^2^ and was used for the testing set. Microarray and RNA-seq data included in our study were normalized, and log2 was transformed.

### Promising Risk Hallmark Selection and Validation

To evaluate the levels of various hallmarks in ccRCC, TCGA gene expression matrix was transformed into a hallmark enrichment score matrix *via* “gsva,” an R package that contained a single-sample gene set enrichment analysis (ssGSEA) algorithm (Barbie et al., 2009). The hallmark gene sets were downloaded from the Molecular Signatures Database^3^. Univariate and multivariate Cox regression analysis was performed to select the promising risk hallmarks, and the hallmark that satisfied previous analysis with the lowest *p*-value was chosen. To validate that the risk hallmark could be a risk factor, we compared ssGSEA score of promising risk hallmarks between normal kidney tissues and tumor tissues. The previous comparison was also done between alive and dead patients in ccRCC. Moreover, we conducted Gene Set Enrichment Analysis (GSEA) between normal kidney tissues and tumor tissues. Furthermore, GSEA and survival analysis were performed in different groups, grouping by median hallmarks enrichment score.

### Genes of Promising Risk Hallmark Further Selection

Based on TCGA gene expression matrix, a scale-free co-expression network was identified, and genes with similar expression patterns were clustered into the same module. Subsequently, the module with the highest correlation to risk hallmarks was identified. To pick out the hub genes from the previous module, gene significance for the module was set as 0.5, and module membership was set as 0.8. Genes that met the previous critical limitation were chosen for further study as promising candidates.

### Prognosis Model Establishment and Validation

The promising gene expression matrix was divided into training sets (80%) and validation sets (20%). The least absolute shrinkage and selection operator (LASSO) Cox regression model was performed to identify the most robust prognostic genes from promising genes. Here, the IL6-JAK-STAT3 pathway-related risk score (IRS) was calculated as follows: IRS = Sum (Expression of the most robust prognostic genes^∗^ their LASSO Cox coefficients). Subsequently, the patients were divided into high-IRS and low-IRS groups based on median IRS. The log-rank test was used to plot the Kaplan–Meier survival curve between these two groups. To determine gene signature accuracy, the receiver operating characteristic (ROC) curve was generated *via* R package “timeROC.” Besides, dataset GSE 40435 from another platform was used to re-evaluate module accuracy. The IRS of dataset was calculated, and it was split in the same manner as previously done. For inaccessible follow-up information, we performed GSEA in different IRS groups and explored the relationship between IRS and pathology grade.

### Further Bioinformatic and Statistical Analyses

Recently, increased focus has been paid to tumor microenvironment and alternative polyadenylation, despite the fact that their functions in ccRCC remain unknown. As a result, we herein estimated the abundance of infiltrating immune cells and predicted tumor purity in different levels of ssGSEA score. To examine the relationship between PD-1 and ssGSEA score, we compared PD-1 expression in high-ssGSEA score and low-ssGSEA score groups. Alternative polyadenylation (APA) is emerging as a new paradigm of post-transcriptional regulation for > 70% of human genes (Elkon et al., 2013). Recently, research has revealed that APA plays a significant role in various cancers (Kang et al., 2020; Zhang et al., 2021). The Cancer 3′ UTR Atlas (Feng et al., 2018) is an online database that, in conjunction with other genomics profiles in cBioPortal, may be used to investigate the functional consequences of APA events in human cancers. We explored APA events of promising genes and their correlation with prognosis in ccRCC.

### Established Nomogram of Combined Signature With Clinical Characteristics and APA

To further enhance prognostic accuracy, signature with clinical characteristics (age, gender, grade, stage, and T) and external risk factor APA of FMNL1 were combined for established nomogram. This process was accomplished by R package “rms.” Here, time-dependent receiver operating characteristic analysis was used to measure the predictive power of different risk factors *via* R package “timeROC,” and the results were compared *via* R package “tidyverse.”

## Results

### The IL6-JAK-STAT3 Pathway Was Identified as a Promising Risk Hallmark

**FIGURE 2:**
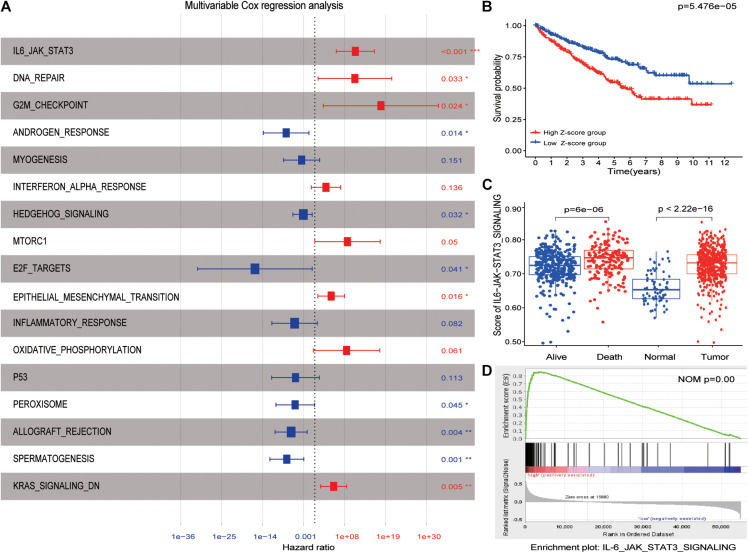
IL6-JAK-STAT3 pathway was identified as candidate risk hallmarks. **(A)** The results of multivariable Cox regression analysis. *p*-values were shown as: *p < 0.05; **p < 0.01; ***p < 0.001. **(B)** The high *Z*-score group had a worse outcome than the low *Z*-score group.**(C)** The enrichment score of the IL6-JAK-STAT3 pathway was significantly different between normal kidney tissue and ccRCC patients. The same difference was also found between alive and dead patients. **(D)** GSEA results between high *Z*-score and low *Z*-score groups.

After performing univariate analysis and multivariate Cox regression analysis in the hallmark enrichment score matrix, the results are shown in Figure 2A. Four hallmarks were the risk factors, while six hallmarks were on the contrary. Here, the IL6-JAK-STAT3 pathway was identified as the promising risk hallmark with the lowest *p*-value. Figure 2B reveals that patients with a high *Z*-score (hallmarks enrichment score) had a worse prognosis than those with a low *Z*-score. More interestingly, the IL6-JAK-STAT3 pathway score in dead patients was higher when compared with alive ones, as displayed in Figure 2C. This phenomenon was also observed between normal kidney tissues and tumor tissues (Figure 2C and Supplementary Figure 1). The GSEA results also demonstrated that the IL6-JAK-STAT3 pathway was functional in a higher *Z*-score group (Figure 2D). All these results demonstrate that the IL6-JAK-STAT3 pathway was the suitable promising risk hallmark.

### A Part of Genes in the Cyan Module Was Selected for Further Analysis

**FIGURE 3:**
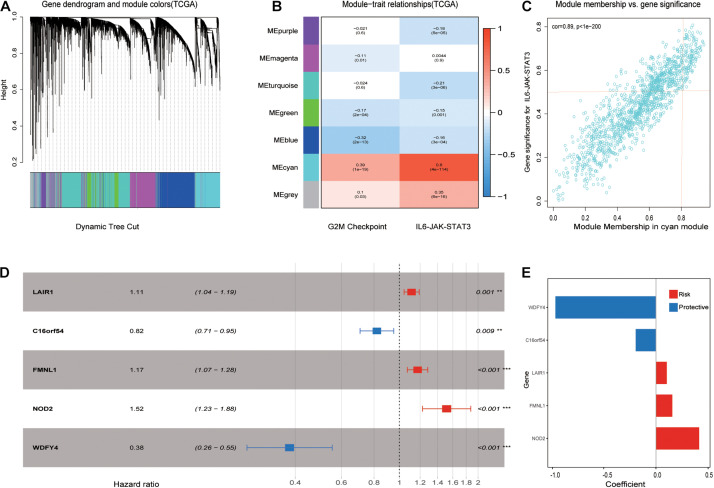
Picked out candidate risk hallmark-related genes and constructed a prognosis model. **(A)** Genes with similar expression patterns were clustered into the same module. **(B)** The relationship between module and clinical traits. The MEcyan module was significant with the IL6-JAK-STAT3 pathway most with a score of 0.8 and a *p*-value of 4e-114. **(C)** A scatterplot of gene significance (GS) for the IL6-JAK-STAT3 pathway vs. Module membership (MM) in the MEcyan module. **(D)** LASSO Cox regression analysis for OS of five pathway-related genes in ccRCC. *p*-values were shown as: **p < 0.01; ***p < 0.001. **(E)** Genes with their coefficient score in LASSP Cox regression analysis.

After performing WGCNA, a scale-free co-expression network was identified. The genes were clustered into seven modules with a different color (Figure 3A). Among these modules, the cyan module exhibits the highest correlation score with the IL6-JAK-STAT3 pathway at 0.8. The score of the *p*-value was 4e–114 (Figure 3B). In addition, module membership vs. gene significance results are shown in Figure 3C, indicating that the cyan module was closely related to the IL6-JAK-STAT3 pathway. A total of 142 genes in the cyan module met the previous limitation and were chosen for further analysis. Subsequently, the least absolute shrinkage and selection operator (LASSO) Cox regression analysis was performed and results were shown in Figures 3D,E.

### Five IL6-JAK-STAT3 Pathway-Related Genes Prognosis Model Establishment

**FIGURE 4:**
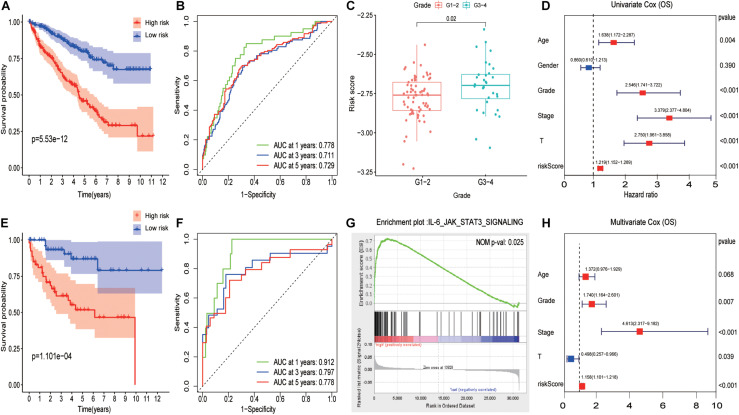
Evaluating pathway-related prognosis model in ccRCC with different perspectives and cohorts. **(A)** A Survival analysis with different risk score groups in the training cohort. **(B)** The ROC curves in the training cohort. **(C)** The boxplot of risk score between Grade 1 to Grade 2 and Grade 3 to Grade 4 in the testing group. **(D)** The results of univariate Cox analysis for OS in ccRCC. **(E)** Survival analysis with different risk score groups in the validation cohort. **(F)** The ROC curves in the validation cohort. **(G)** GSEA results between high-risk score and low-risk score groups. **(H)** The results of multivariate Cox analysis for OS in ccRCC.

Subsequently, ccRCC samples from TCGA were randomly divided into train cohort and validation cohort *via* R package “caret” in ratios of 8:2. Using R package “survival and survminer,” the signature was established and the IL6-JAK-STAT3 pathway-related risk score (IRS) was calculated as follows: IRS = (0.11^∗^ LAIR1) + (–0.20^∗^ C16orf54) + (0.16^∗^ FMNL1) + (0.42^∗^ NOD2) + (–0.97^∗^ WDFY4). As Figure 4A displays, patients with a high IRS had a worse outcome than those with low IRS in the training cohort. The predictive performance results of IRS are illustrated in Figure 4B, and the area under the curve reached 0.778 at 1 year, 0.711 at 3 years, and 0.729 at 5 years. Consistently, the results of the Kaplan–Meier curve and time-dependent ROC curves in the inside validation cohort indicated a good performance of the signature model (Figures 4E,F). Previously, it was tested on a different platform, cohort GSE 40435. Due to a lack of follow-up data, we compared IRS in different grades, and the results presented in Figure 4C demonstrated that patients had a higher IRS as the grade elevated. The GSEA analysis indicated that the IL6-JAK-STAT3 pathway functioned on a higher IRS group as Figure 4G shown.

Univariate and multivariate Cox regression analyses showed that IRS was an independent prognostic predictor with a hazard ratio of 1.158. More intriguingly, pathology grade and tumor stage behave as independent risk factors among various factors while T did not (Figures 4D,H).

### APA of Pathway-Related Genes Was Related to Prognosis in ccRCC

**FIGURE 5:**
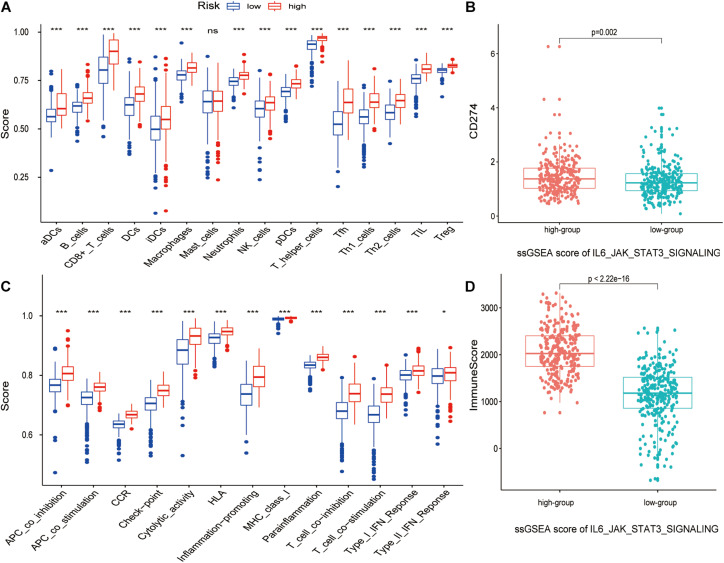
The relation between the IL6-JAK-STAT3 pathway and tumor microenvironment. *p*-values were shown as: ns, not significant; *p < 0.05; ***p < 0.001. **(A)** The boxplot of ssGSEA scores with 16 immune cells in different risk score groups. **(B)** The boxplot of ssGSEA scores with 13 immune-related functions in different risk score groups. **(C)** The boxplot of CD274 expression in different ssGSEA scores of IL6-JAK-STAT3 pathway groups. **(D)** The boxplot of the immune score in different ssGSEA scores of IL6-JAK-STAT3 pathway groups.

**FIGURE 6:**
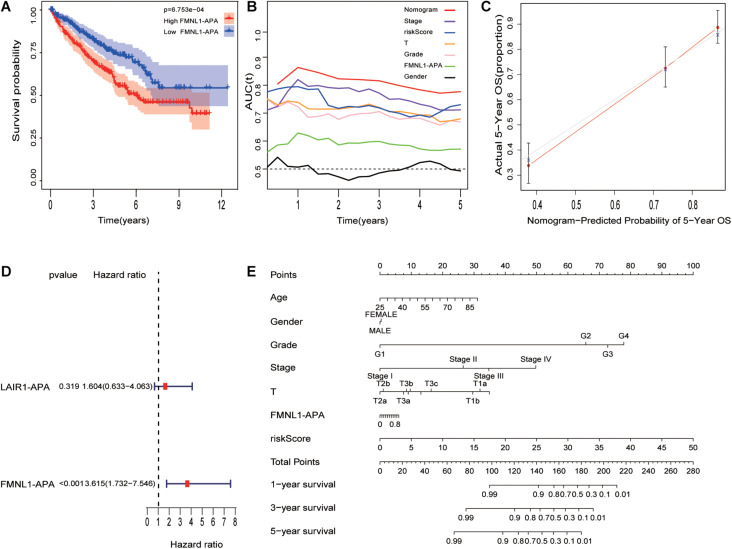
Combination of risk model and clinicopathological features (including APA of genes) improves risk stratification and survival prediction. **(A)** Different FMNL1-APA was closely related to prognosis in ccRCC. **(B)** tROC analysis demonstrated that the nomogram was the most stable and powerful predictor for OS in ccRCC among all clinical variables. **(C)** Calibration analysis of nomogram. **(D)** The univariate analysis revealed that FMNL1-APA was related to prognosis in ccRCC. **(E)** The nomogram was built based on all clinical variables.

The boxplot in Figures 5A,C revealed the difference between immune cells and immune-related functions or pathways in different IRS groups. The score of diverse immune cell subpopulations was higher in high IRS groups except for Mast cells, while immune-related functions or pathways were all more active. Similarly, CD274 expression and immune score were upregulated in higher IRS groups (Figures 5B,D). Figures 6A,D show that APA of FMNL1 could be an independent risk factor, implying that a higher APA of FMNL1 resulted in a worse outcome.

### The Nomogram Improves the Predictive Accuracy of Prognostic

As Figure 6E shows, a nomogram was built with HRS and other clinical characteristics. To evaluate nomogram accuracy, calibration analysis was performed, and the results in Figure 6C suggest high correctness. Compared with other characteristics, the nomogram (Figure 6B) has the strongest and most stable survival predictive ability, and the average AUC is higher than 0.8, much better than traditional characteristics (pathological grade and tumor stage). This means that the nomogram can significantly enhance the ability to predict prognosis.

## Discussion

The IL6-JAK-STAT3 pathway had a vast influence on tumors with various biological progress, such as migration, invasion, and angiogenesis (Ni et al., 2020; Pan et al., 2020; Siersbaek et al., 2020; Tung et al., 2020). Therefore, targeting the IL6-JAK-STAT3 pathway was a promising treatment correlated with multifaceted mechanisms. Numerous new IL-6/JAK/STAT3 pathway inhibitors have been identified to date and are currently undergoing preclinical and/or clinical evaluation (Oh et al., 2015; Liu et al., 2016), whereas few studies were reported on IL6-JAK-STAT3 pathway signature in ccRCC. Considering the IL6-JAK-STAT3 pathway as a complex gene network, a scale-free co-expression network was constructed, and genes in the network that significantly correlated with the targeted pathway were used to establish the model, rather than relying on any individually identified “IL6-JAK-STAT3 pathway-related genes” from literature. In addition, the traditional prognostic system was combined with the model to optimize the clinical routine.

This study translated the gene expression matrix into a hallmark enrichment score matrix using ssGSEA algorithm. Subsequently, the IL6-JAK-STAT3 pathway was identified as promising risk hallmarks *via* univariate and multivariate Cox regression analysis while the performed WGCNA selected the genes closely related to hallmarks. Besides, a LASSO Cox regression model was constructed, and IRS was calculated concurrently. An inside validation cohort and test cohort from other platforms identified the prognosis value of the model. Considering APA and tumor microenvironment are tightly closed with tumor, we preliminary explored its relation with the IL6-JAK-STAT3 pathway. Besides, improving the predictive power and accuracy of prognosis in ccRCC was accomplished by establishing a nomogram including IRS with other available traditional clinicopathological features. Calibration analysis revealed that prediction was extremely close to actual survival. More interesting, the AUC(t) of the nomogram fluctuated around 0.8, much better than other predictive models at different time points during follow-up.

A part of genes we identified was studied in many reports. Mey et al. (2019) revealed that NOD2 expression is upregulated in ccRCC with a mouse xenograft model. Xu et al. (2017) demonstrated that NO_2_ might be a biomarker for the survival of kidney cancer patients. This phenomenon was also observed in LAIR1 (Jingushi et al., 2019). Besides, Peng et al. (2020) claimed that collagen promotes anti-PD-1/PD-L1 resistance in cancer through LAIR1-dependent CD8^+^ T cell exhaustion, similar to Xu et al.’s findings (Xu et al., 2020). The reports about FMNL1 extensively focused on its role in aggressiveness of many tumors, such as nasopharyngeal carcinoma, leukemia, and ccRCC (Favaro et al., 2013; Chen et al., 2018; Zhang et al., 2020), while WDFY4 extensively focused on autoimmune diseases (Kochi et al., 2018; Yuan et al., 2018; Zhu et al., 2020). Unlike previous genes, the reports about c16 or f54 were limited and still need further investigation.

Despite the good performance of the nomogram, some limitations in our study remain, requiring further investigation. First, the study was based on public datasets, requiring further verification in our own clinical cohort. Second, as a retrospective study, it needs further validation in a larger prospective cohort. Finally, further experimental studies are needed to explore the roles that five pathway-related genes play in ccRCC.

## Conclusion

We established an IL6-JAK-STAT3 pathway-related gene prognosis model to predict the outcomes in ccRCC patients. Based on this model, we combined traditional clinicopathological features in advance and constructed a nomogram with more stable and accurate survival predictive ability. Moreover, this study would provide new insights for guiding individualized treatment of ccRCC patients.

## Data Availability Statement

Publicly available datasets were analyzed in this study. This data can be found here: TCGA and NCBI database.

## Author Contributions

CZ and ZW conceived and designed the study, collected the data, performed the analysis, and wrote the manuscript. XY, ZL, YR, GW, HZ, MQ, and CX contributed to data and analysis tools. JC and JL helped to revise the manuscript. CS was guidance and advice. All authors contributed to the article and approved the submitted version.

## Conflict of Interest

The authors declare that the research was conducted in the absence of any commercial or financial relationships that could be construed as a potential conflict of interest.

## Publisher’s Note

All claims expressed in this article are solely those of the authors and do not necessarily represent those of their affiliated organizations, or those of the publisher, the editors and the reviewers. Any product that may be evaluated in this article, or claim that may be made by its manufacturer, is not guaranteed or endorsed by the publisher.

## Footnotes

1
https://www.cancer.org/

2
http://www.ncbi.nlm.nih.gov/geo/

3
http://www.gsea-msigdb.org/gsea/msigdb
