# Supplementary figures and images for: Development and Validation of an IL6/JAK/STAT3-Related Gene Signature to Predict Overall Survival in Clear Cell Renal Cell Carcinoma
Source: Front Cell Dev Biol. 2021 Sep 29;9:686907. doi: 10.3389/fcell.2021.686907 (PMC8511427; doi:10.3389/fcell.2021.686907)

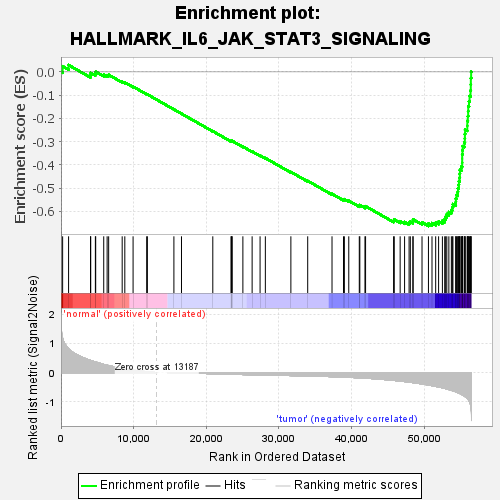

Supplement: Supplementary file 1 [file Image_1.PNG]
